# High prevalence of multidrug-resistant and ESBL-producing bacteria in a primary healthcare facility in Accra, Ghana: A cross-sectional study

**DOI:** 10.1371/journal.pgph.0004991

**Published:** 2025-08-06

**Authors:** Frank Twum Aboagye, Mawutor Kwame Ahiabu, Hannah Segbefiah Akahoho, Emmanuel Armah, Samuel G. Anang, Naa A. S. Amarteifio, Christabel Amoo, Abena Konadu Owusu-Senyah Enninful, Queenstar Dedei Quarshie, Naa Adjeley Kuma, Judith Wayo, Kantanka Addo-Osafo

**Affiliations:** 1 Biomedical and Public Health Research Unit, Water Research Institute, Council for Scientific and Industrial Research, Accra, Ghana; 2 The Graduate School, Augusta University, Augusta, Georgia, United States of America; 3 Department of Genetics and Biochemistry, Clemson University, Clemson, South Carolina, United States of America; 4 Department of Medical Microbiology, University of Ghana Medical School, University of Ghana, Korle Bu, Accra, Ghana; 5 Department of Medical Laboratory Technology, Faculty of Applied Sciences, Accra Technical University, Accra, Ghana; Universiti Kebangsaan Malaysia, MALAYSIA

## Abstract

Antimicrobial resistance (AMR) is a global public health crisis, particularly in low- and middle-income countries where empirical antibiotic use is common. Data on bacterial resistance patterns in primary healthcare settings remain limited in Ghana, impeding effective treatment guidelines and infection control measures. This cross-sectional study, conducted at Shalina Health Centre in Accra, Ghana, between May and November 2024, examined bacterial infections among 156 systematically sampled patients. Clinical specimens underwent bacterial culture, identification via Gram staining and biochemical tests, and antibiotic susceptibility testing using the Kirby-Bauer disc diffusion method. Extended-spectrum beta-lactamase (ESBL) production was assessed per Clinical and Laboratory Standards Institute (CLSI) guidelines. Statistical analysis included prevalence estimation with 95% confidence intervals and subgroup comparisons using ANOVA and Chi-square tests. Bacterial infections were detected in 62.20% [97/156] of participants, with *Escherichia coli* [43.30%], *Staphylococcus aureus* [21.65%], and *Klebsiella pneumoniae* [19.59%] as the most prevalent pathogens. Gram-negative bacteria accounted for 75.26% of isolates. Females exhibited a significantly higher infection rate [69.64%] than males [43.18%] [p = 0.002]. Multidrug resistance (MDR) was widespread [97.94%], and 76.06% of Enterobacteriaceae isolates were ESBL producers. Resistance to fluoroquinolones, cephalosporins, and beta-lactams was particularly high among Gram-negative isolates. The study highlights the high burden of multidrug-resistant bacteria in primary healthcare settings, with significant resistance to key antibiotics. These findings emphasize the urgent need for enhanced antimicrobial stewardship, evidence-based prescribing practices, and strengthened infection prevention measures. Surveillance in non-tertiary healthcare facilities is crucial to mitigate AMR transmission and optimize treatment protocols in Ghana and similar settings.

## Introduction

Bacterial infections remain a significant challenge to healthcare systems worldwide, particularly in low- and middle-income countries with limited resources and high patient loads [1,2]. These infections contribute substantially to illness and death, especially among hospitalized patients, individuals with weakened immune systems, and the elderly [3]. The discovery of antimicrobials revolutionized modern medicine, providing effective treatments against bacterial infections [4]. However, several factors, including decades of widespread use and abuse of antibiotics, have contributed to bacterial resistance rendering them ineffective [5]. The increasing resistance to commonly used antimicrobials complicates treatment, prolongs recovery times, and raises healthcare costs [6]. Antimicrobial resistance (AMR) has gained significant notoriety as a major global health threat, leading to prolonged hospital stays, higher medical expenses, and increased mortality [7,8]. The impact is particularly severe in Sub-Saharan Africa, where access to advanced diagnostics and effective treatments is often limited [9]. In 2019, an estimated 1.27 million deaths were attributed to bacterial AMR globally, with Western Sub-Saharan Africa recording the highest number of AMR-related deaths [10]. These statistics are cause for concern as it is agreed that understanding bacterial resistance patterns is critical for developing treatment guidelines and public health strategies.

Monitoring bacterial prevalence and resistance patterns is essential for improving antimicrobial stewardship programs, guiding empirical treatment, and controlling the spread of resistant infections within hospitals and communities. In Ghana, most studies on bacterial resistance have been conducted in tertiary hospitals, leaving a gap in data from health centres that serve as the first point of care before referrals to higher-level facilities [11,12]. We believe that primary healthcare facilities play a key role in infection prevention and serve as entry points for patients who may later require hospitalization, contributing to the overall burden of healthcare-associated infections. As such, understanding AMR patterns in these facilities is crucial for the fight against AMR. Insights from this study can inform infection control protocols, helping to reduce AMR transmission from primary care to tertiary hospitals, ultimately strengthening national infection prevention strategies.

According to Mancusso and colleagues, clinical management of bacterial pathogens responsible for urinary tract, respiratory and bloodstream infections has become increasingly difficult due to antibiotic resistance [13]. Notably, *Escherichia coli* and *Klebsiella pneumoniae*, both infectious species of the Enterobacteriaceae family, are of particular concern. These bacterial species have been shown to have extensive resistance to multiple antimicrobials, including extended-spectrum beta-lactamases (ESBLs) [14]. This study examines the prevalence and resistance profiles of *E. coli and K. pneumoniae* and other bacterial pathogens at a primary healthcare facility (Shalina Health Centre) with aims to provide data that can inform treatment guidelines and infection control measures in similar settings.

## Methodology

### Ethics statement

The study was approved by the Ethical Review Committee of the Medical Laboratory Technology Department, Accra Technical University (Protocol No.: ATU/MLT/ET/01210943B/01210228B/01200786B/2023–2024). Written and informed consent was obtained from all participants, with assent secured from guardians for minors (< 18 years). Participants were assured of confidentiality and data security, and all study procedures adhered to ethical guidelines for human research per the Declaration of Helsinki. The written informed consent explicitly covered the inclusion of additional bacterial testing beyond the primary clinical suspicion, ensuring participants were aware of extended diagnostic procedures. All patient data were anonymized, and unique identification codes were assigned to protect confidentiality. Data were securely stored in password-protected systems, accessible only to authorized researchers.

### Study design and population

This cross-sectional study was conducted at Shalina Health Centre, a private healthcare facility in Accra, Ghana. Clinical samples were obtained from both inpatient and outpatient departments to capture a broad range of bacterial infections across different levels of care. The centre was selected for its accessibility to neighbouring urban and peri‑urban populations and its function as a first point of contact for both routine and urgent medical needs. Clinical specimens were collected from patients attending inpatient and outpatient services to ensure representation of infections encountered across varying levels of care. All relevant data describing the study participants have been provided in accordance with the data sharing policy of the journal.

Shalina Health Centre maintains 20 beds and provides continuous outpatient services. It manages approximately 150–200 patient visits per day, encompassing general medical consultations, maternal and child health checks, and minor surgical cases. The facility draws attendees from Korle Bu, Dansoman, Jamestown and parts of Kaneshie, reflecting a diverse urban catchment. Its patient volume and community reach make it an appropriate setting for assessing antimicrobial resistance in a primary care context. To account for seasonal variations in bacterial infections and to allow for sufficient sample collection to analyse resistance patterns effectively, the study was conducted between the period of 21^st^ May and 5^th^ November 2024.

### Eligibility criteria

#### Inclusion criteria.

Patients were eligible for inclusion if they presented with symptoms suggestive of bacterial infections, such as fever, dysuria, wound discharge, or respiratory distress. To ensure reliable antimicrobial resistance data, only patients who had not received antibiotic treatment within 72 hours before specimen collection was included. Only individuals who provided informed consent, or in the case of minors, assent from a guardian, were enrolled as participants. Complete medical records with sufficient clinical and demographic data were required for inclusion.

#### Exclusion criteria.

Patients were excluded if they had received antibiotic treatment within 72 hours before sample collection, as prior exposure could alter bacterial culture and susceptibility results. Individuals diagnosed with non-bacterial infections (e.g., viral, or fungal infections) were also excluded to maintain the focus on bacterial pathogens. Patients with chronic infections requiring long-term treatment, such as tuberculosis, were not included, as their resistance patterns might not reflect acute bacterial resistance trends. Specimens that were contaminated or insufficient for analysis were excluded. Additionally, patients with incomplete medical records, and those who declined consent, were not eligible.

### Sample size and sampling technique

The sample size was estimated using the Cochrane formula for cross-sectional studies. The proportion was obtained based on a previous prevalence of bacterial infection (11.3%) reported by Tabiri et al. [15] with a 95% confidence level and a 5% margin of error.


$$n=\frac{1.96^{2}\times 0.113(1-0.113)}{0.05^{2}}\Rightarrow n=155$$

A final sample size of 156 patients was selected based on practical feasibility while ensuring adequate statistical power, allowing for the detection of significant differences in bacterial prevalence and resistance patterns.

A systematic random sampling technique was used, where every third patient presenting with suspected bacterial infection or requesting a culture and sensitivity test during the study period was included after obtaining informed consent. Although a systematic random sampling method was utilized to ensure representativeness, potential selection biases were recognized. To mitigate these biases, sample collection was performed across different shifts (morning, afternoon, and evening) and included patients from both outpatient and inpatient departments to enhance diversity in the study population. This sample size was deemed sufficient to detect significant differences in resistance patterns across bacterial species, MDR versus non-MDR isolates, and Gram-positive versus Gram-negative bacteria with adequate statistical power. This approach ensured fair representation of bacterial isolates across different patient demographics.

### Sample collection

Clinical specimens, including urine, blood, wound swabs, high vaginal swabs (HVS), were collected from patients presenting with bacterial infection symptoms. Samples were obtained under sterile conditions by trained personnel following standard clinical protocols to prevent contamination. Urine samples were collected using the clean-catch midstream method, while wound and vaginal swabs were taken using sterile swabs. All specimens were promptly transported to the hospital’s microbiology laboratory for analysis. Sample processing was initiated within an hour of collection to preserve bacterial viability and prevent overgrowth of contaminants.

All specimens were submitted to the hospital’s microbiology laboratory for culture and antimicrobial susceptibility testing (AST). These procedures form part of the facility’s routine diagnostic services and were requested at the discretion of attending clinicians. During the study period, laboratory testing was integrated into standard clinical care and not conducted solely for research purposes. Due to the time required for laboratory analysis, initial treatment was often empirical. However, where necessary, particularly for inpatients or patients who returned for follow-up, treatment was revised based on AST results provided by the laboratory.

Standard biosafety protocols were followed during sample collection to ensure patient safety, particularly for blood draws and other invasive procedures. Infection control measures, including the use of sterile techniques and proper disposal of biohazardous materials, were strictly adhered to. Standard biosafety measures were implemented to ensure patient comfort and safety during sample collection, especially for blood draws and invasive procedures. Infection control measures, including the use of sterile techniques and proper disposal of biohazardous materials, were strictly adhered to.

### Laboratory analysis

#### Specimen processing and bacteria identification.

Upon arriving at the laboratory, samples were inoculated onto blood agar, MacConkey agar (Biomark Laboratories, India; Product Code: B1222), and chocolate agar (Biomark Laboratories, India; Product Code: B980) based on the type of specimen. Cultures were incubated at 37°C for 24–48 hours, and bacterial growth was monitored. Isolates were identified based on colony morphology, Gram staining, and biochemical tests, such as lactose fermentation, oxidase, citrate, motility, indole, urease, and triple sugar iron (TSI) tests. To differentiate *Enterococcus faecalis* from *Enterococcus faecium*, pyruvate, aesculin bile agar, and 6.5% sodium chloride broth were used (S1 Table). For Gram-negative rods, the Analytical Profile Index (API) 20E system was utilized when conventional methods did not yield definitive identification.

#### Antibiotic susceptibility testing (AST).

Antimicrobial susceptibility testing was performed using the Kirby-Bauer disc diffusion method [16]. Bacterial suspensions were adjusted to 0.5 McFarland standard and inoculated onto Mueller-Hinton agar (Biomark Laboratories, India; Product Code: B263) using sterile cotton swabs to ensure uniform bacterial growth. Antibiotic discs were applied using a disc dispenser, and plates were incubated at 37°C for 18–24 hours. The zones of inhibition were measured using a digital vernier calliper and interpreted according to Clinical and Laboratory Standards Institute [17] guidelines.

The following antibiotic discs (Biomark Laboratories, Pune, India) were selected based on their relevance to commonly prescribed antibiotics in Ghana, local resistance patterns, and WHO’s priority list of antimicrobials for surveillance. Specific gram-negative [Tetracycline (10 µg), Meropenem (10 µg), Cefuroxime (30 µg), Vancomycin (30 µg), Amikacin (30 µg), Cotrimoxazole (25 µg), Gentamicin (10 µg), Ampicillin (10 µg), Ciprofloxacin (5 µg), Ceftriaxone (30 µg) and Chloramphenicol (10 µg)] and gram-positive [Cloxacillin (5 µg), Ampicillin (10 µg), Erythromycin (5 µg), Ciprofloxacin (5 µg), Augmentin (30 µg), Cotrimoxazole (25 µg), Gentamicin (10 µg), Vancomycin (30 µg), Meropenem (10 µg), Tetracycline (30 µg) and Penicillin (1.5 µg)] antimicrobial discs (Biomark Laboratories, Pune, India) were selected for gram-negative and gram-positive isolates. Also, isolates from HVS and urine were tested using UTI panel of antibiotics from Biomark Laboratories (Pune, India): [Ciprofloxacin (5 µg), Norfloxacin (20 µg), Ceftazidime (20 µg), Amikacin (30 µg), Nitrofurantoin (300 µg), Gentamicin (10 µg), Ceftriaxone (30 µg), Levofloxacin (5 µg), Augmentin (30 µg), Tetracycline (30 µg), Piperacillin (20 µg) and Nalidixic acid (30 µg)]. Multidrug resistance (MDR) was defined as resistance to at least one agent in three or more antibiotic categories, following CLSI 2021 criteria [17].

#### Extended-spectrum beta-lactamase (ESBL) screening.

All Enterobacteriaceae isolates were screened for ESBL production using Ceftazidime (30 µg) and Cefotaxime (30 µg) discs. Isolates resistant to either antibiotic was further tested using Cefpodoxime (10 µg) alone and Cefpodoxime-clavulanic acid (10/1 µg). ESBL production was confirmed if there was a ≥ 5 mm increase in the zone of inhibition with the combination disc.

### Quality control

Quality control for AST was maintained through internal verification by duplicate testing of selected isolates and external benchmarking against CLSI-defined breakpoints to ensure accuracy and reproducibility of susceptibility results. Reference bacterial strains were used to validate culture, identification, and AST procedures. *Escherichia coli* ATCC 25922 and *Staphylococcus aureus* ATCC 29213 were included for susceptibility testing. At the same time, *Klebsiella pneumoniae* ATCC 700603 (ESBL-positive) and *Escherichia coli* ATCC 25922 (ESBL-negative) were used as controls for ESBL detection. To ensure reliability of ESBL detection, randomly selected isolates were re-tested for ESBL production as an internal validation step. Additionally, external benchmarking was performed by cross-checking results with CLSI-defined ESBL confirmation criteria.

### Data and statistical analysis

Data obtained in the study was entered into Microsoft Excel (Microsoft Corp., Washington, USA). Any missing or incomplete data were assessed, and cases with critical missing values were excluded from analysis to maintain data integrity. After cleaning and validation of the data, it was imported into Statistical Package for the Social Sciences (SPSS) version 27 (IBM Corp., Armonk, NY, USA) and GraphPad Prism 9.0 (GraphPad Software Inc., Boston, USA) was used for analysis. For categorical variables, frequencies and proportions were also calculated. Participants were categorised into three age groups: ≤ 30 years, 31–59 years, and ≥60 years. These categories were chosen to reflect the natural distribution of patients presenting at the facility and to enable meaningful statistical analysis within each group as done elsewhere [18], given the sample size. Prevalence of bacterial infection was determined as the ratio of the number of participants with bacterial growth after culture to the total number of participants. Prevalence was reported with its corresponding 95% confidence interval. Statistical comparison between subgroups of categories was evaluated by the ANOVA and Chi-square test where appropriate. To account for the risk of Type I error due to multiple comparisons, Bonferroni correction was applied where necessary to adjust p-values for multiple hypothesis testing, ensuring robust statistical inference.

## Results

### Demographic profile of study participants

Of the 156 participants examined, 71.80% [n = 112] were females and 28.20% [n = 44] were males. Also, 49.40% [n = 77] were aged ≤ 30 years and 12.10% [n = 19] were aged ≥ 60 years. As shown in Table 1, majority of the specimen submitted for assessment was urine [n = 115, 73.70%], followed by HVS [n = 24, 15.40%]. Only two blood specimens were submitted for assessment [1.30%].

**Table 1:** Demographic characteristics of study participants.

Characteristics	Frequency	Percentage	p-value
**Gender**			< 0.001
Male	44	28.20	
Female	112	71.80	
**Age (years)**			< 0.001
≤ 30	77	49.40	
31 – 59	60	38.50	
≥ 60	19	12.10	
**Specimen Submitted**			< 0.001
Blood	2	1.30	
High Vaginal Swab (HVS)	24	15.40	
Stool	11	7.00	
Urine	115	73.70	
Wound	4	2.60	

p-values were generated using one-sample chi-square test.

### Prevalence of bacterial infection

**Fig 1:**
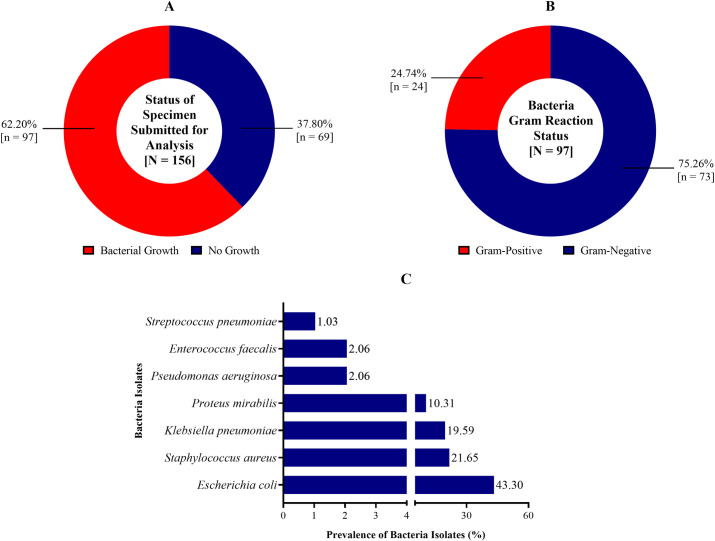
Prevalence of bacterial infection among study participants [A: Overall Prevalence; B = Gram reaction status of bacteria; C = Specific bacteria specie isolated].

The prevalence of bacterial infection was 62.20% [97/156, CI_95_: 54.34 – 69.41] (Fig 1A). Bacteria characterisation using Gram-Reaction showed that 24.74% [24/97, CI_95_: 17.24 – 34.21] of the isolates were Gram-positive and 75.26% [73/97, CI_95_: 65.79 – 82.76] were Gram-negative (Fig 1B). Statistically, the prevalence of Gram-negative bacteria differed significantly from that of the Gram-positive bacteria [χ^2^ = 49.259, p < 0.001]. Seven different bacteria pathogens were identified (Fig 1C). 43.30% [42/97, CI_95_: 33.87 – 53.25] of the isolates were identified to be *Escherichia coli*, while 21.65% [21/97, CI_95_: 14.63 – 30.87] were classified as *Staphylococcus aureus* and 19.59% [19/97, CI_95_: 12.93 – 28.61] were also identified as *Klebsiella pneumoniae*. As shown in Fig 1C, the prevalence of *Proteus mirabilis*, *Pseudomonas aeruginosa*, *Enterococcus faecalis* and *Streptococcus pneumoniae* was 10.31% [10/97, CI_95_: 5.73 – 17.96], 2.06% [2/97, CI_95_: 0.64 – 7.18], 2.06% [2/97, CI_95_: 0.64 – 7.18] and 1.03% [1/97, CI_95_: 0.25 – 5.55] respectively.

### Demographic characteristics and specimen types with bacterial infection

**Fig 2:**
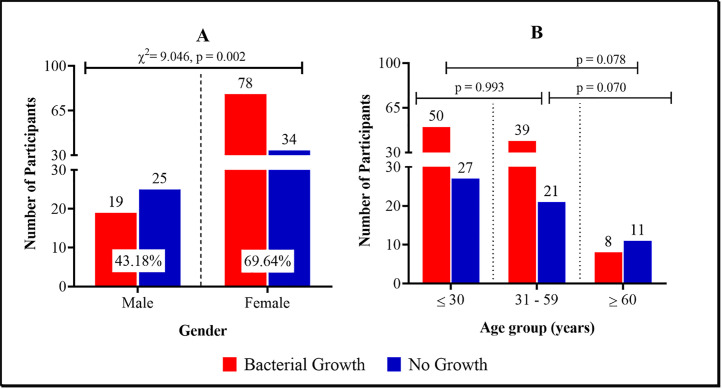
Prevalence of bacterial infection stratified by [A] gender and [B] age.

Fig 2 gives a detailed description of the distribution bacterial infection among the study participants. As shown in Fig 2A, the prevalence of bacterial infection among the male participants was 43.18% [19/44, CI_95_: 29.64 – 57.85] and among the female participants, the prevalence reported was 69.64% [78/112, CI_95_: 60.56 – 77.39]. Statistically, the prevalence of bacterial infection in males and females differed significantly [χ^2^ = 9.046, p = 0.002] (Fig 2A). Age-wise, the prevalence of bacterial infection among participants aged ≤ 30 years was 64.93% [50/77, CI_95_: 53.76 – 74.65] and 65.00% [39/60, CI_95_: 52.31 – 75.84] among the 31 – 59-year-olds (Fig 2B). Furthermore, the prevalence reported among the participants aged *≥ 60* years was 42.11% [8/19, CI_95_: 23.06 – 63.95]. The prevalence of bacterial infection did not differ significantly [p > 0.05] across the age groups as shown in Fig 2B.

Table 2 presents the prevalence of the isolated bacterial pathogens among the study participants. The most prevalent pathogen in both the male [n = 8, 18.18%] and female [n = 34, 30.36%] participants was *Escherichia coli*. However, there was no significant difference in the prevalence of *Escherichia coli* between the male and female participants [p > 0.05]. Nonetheless, the prevalence of *Proteus mirabilis* among females differed significantly from that of the male participants [p < 0.05]. The prevalence of *Enterococcus faecalis, Klebsiella pneumoniae, P. aeruginosa* and *Streptococcus pneumoniae* in females were higher than in males as shown in Table 2. However, the prevalence of *Staphylococcus aureus* in this study was not significantly [p < 0.05] higher in males [n = 6, 13.63%] than in females [n = 15, 13.39%]. As shown in Table 2, participants aged ≤ 30 years [n = 21, 27.27%] and 31 – 59 years [n = 18, 30.00%] were predominantly infected with *Escherichia coli*. However, the most prevalent bacteria isolated from specimen provided by participants aged ≥ 60 years was *Klebsiella pneumoniae* [n = 4, 21.05%]. Statistically, there was no significant difference in the prevalence of the isolated bacteria across the age groups [p > 0.05].

**Table 2:** Distribution of isolated bacterial pathogens among the study participants.

	N	*E. faecalis* n [%]	*E.* *coli* n [%]	*K. pneumoniae* n [%]	*P. mirabilis* n [%]	*P. aeruginosa* n [%]	*S. aureus* n [%]	*S. pneumoniae* n [%]
**Gender**								
Male	44	0 [0.00]	8 [18.18]	5 [11.36]	0^a^ [0.00]	0 [0.00]	6 [13.63]	0 [0.00]
Female	112	2 [1.78]	34 [30.36]	14 [12.50]	10^a^ [8.93]	2 [1.78]	15 [13.39]	1 [0.89]
**Age group (years)**								
* ≤ 30*	77	1 [1.29]	21 [27.27]	9 [11.69]	8 [10.39]	0 [0.00]	10 [12.99]	1 [1.29]
*31 - 59*	60	1 [1.67]	18 [30.00]	6 [10.00]	2 [3.33]	1 [1.67]	11 [18.33]	0 [0.00]
59 – 84	19	0 [0.00]	3 [15.79]	4 [21.05]	0 [0.00]	1 [5.26]	0 [0.00]	0 [0.00]

*Column values with the same superscript (a) differ significantly at p < 0.05.*

*The values reported are number of positives [n] and the corresponding proportion of the total of the subgroup [%]*.

The distribution of microorganisms isolated from various clinical specimens is shown in Table 3. A total of 97 specimens showed bacteria growth, comprising 20 [20.62%] high vaginal swabs (HVS), 73 [75.25%] urine samples, and 4 [4.13%] wound swabs. Out of the 20 HVS with microbial growth, 9 [45.00%] were identified as *Proteus mirabilis*, 7 [35.00%] were classified as *Staphylococcus aureus* and 3 [15.00%] were identified as *Klebsiella pneumoniae.* Only 1 [5.00] HVS specimen had *Escherichia coli* growth (Table 3). Of the 73-urine specimen with bacteria growth, *Escherichia coli* was the dominate bacteria pathogen isolated [n = 41, 56.16%] and one (1) isolate each of *Proteus mirabilis* [1.37%] and *Streptococcus pneumoniae* [1.37%] were isolated from the urine of the participants. As shown in Table 3, 16 [21.92%] and 10 [13.70%] of the isolates from the urine specimen were identified as *Klebsiella pneumoniae* and *Staphylococcus aureus* respectively. It is worth noting that only *Staphylococcus aureus* was identified in the 4 [100.00] wound swabs that had bacteria growth (Table 3).

**Table 3:** Distribution of isolated specific bacteria isolates by specimen submitted.

Pathogen	HVS 20 [20.62]	Urine 73 [75.25]	Wound 4 [4.13]
*Enterococcus faecalis*	0 [0.00]0	2 [2.74]	0 [0.00]
*Escherichia coli*	1 [5.00]0	41 [56.16]	0 [0.00]
*Klebsiella pneumoniae*	3 [15.00]	16 [21.92]	0 [0.00]
*Proteus mirabilis*	9 [45.00]0	1 [1.37]	0 [0.00]
*Pseudomonas aeruginosa*	0 [0.00]0	2 [2.74]	0 [0.00]
*Staphylococcus aureus*	7 [35.00]	10 [13.70]	4 [100.00]
*Streptococcus pneumoniae*	0 [0.00]	1 [1.37]	0 [0.00]

*Values reported are number of positives and percentage of specimen positive for the microorganism*. *HVS: High Vaginal Swab*.

### Antibiotic resistance pattern of bacterial isolates

#### Resistance profile of bacteria isolate against gram positive antibiotic panel.

*Staphylococcus aureus* was the only gram-positive bacteria isolated from wound swab and HVS. As shown in Table 4, all the *Staphylococcus aureus* isolates were resistant to Cloxacillin (5 µg) [n = 11, 100.00]. Also, 90.91% [10/11] of the isolates were resistant to Erythromycin (5 µg), while 81.82% [9/11] and 72.73% [8/11] were resistant to Meropenem (10 µg) and Ciprofloxacin (5 µg) respectively. Out of 11 *Staphylococcus aureus* isolates tested against Augmentin (30 µg), Ampicillin (10 µg), Penicillin (1.5 µg), Vancomycin (30 µg) and Tetracycline (30 µg), 90.91% [n = 10], 36.36% [n = 4], 90.91% [n = 10], 54.54% [n = 6] and 72.73% [n = 8] were resistant respectively as shown in Table 4. However, all the eleven (11) *Staphylococcus aureus* isolates tested against Gentamicin (10 µg) were susceptible to the antibiotic (Table 4).

**Table 4:** Resistance profile of Gram-positive isolates against Gram-positive antibiotic panel.

Antibiotics	*S. aureus* [N = 11]	*E. faecalis* [N = 0]	*S. pneumoniae* [N = 0]
Cloxacillin (5 µg)	11 [100.00]	─	─
Ampicillin (10 µg)	4 [36.36]	─	─
Erythromycin (5 µg)	10 [90.91]	─	─
Ciprofloxacin (5 µg)	8 [72.73]	─	─
Augmentin (30 µg)	10 [90.91]	─	─
Cotrimoxazole (25 µg)	9 [81.82]	─	─
Gentamicin (10 µg)	0 [0.00]	─	─
Vancomycin (30 µg)	6 [54.54]	─	─
Meropenem (10 µg)	9 [81.81]	─	─
Tetracycline (30 µg)	8 [72.73]	─	─
Penicillin (1.5 µg)	10 [90.91]	─	─

*Values reported are number of resistant isolates and proportion of the total number of isolates tested against the antibiotic [N]; “–” means isolate not tested against the antibiotic*.

#### Resistance profile of bacteria isolate against gram negative antibiotic panel.

Gram-negative bacteria isolated from HVS in this study were *Escherichia coli* and *Klebsiella pneumoniae.* As shown in Table 5, *Escherichia coli* was resistant to Tetracycline (10 µg), Meropenem (10 µg), Cefuroxime (30 µg) and Vancomycin (30 µg). However, *Escherichia coli* susceptible to the action of Amikacin (30 µg), Cotrimoxazole (25 µg), Gentamicin (10 µg), Ampicillin (10 µg), Ciprofloxacin (5 µg), Ceftriaxone (30 µg) and Chloramphenicol (10 µg). It is worth noting that *Klebsiella pneumoniae* was not susceptible to any of these antibiotics. All the *Klebsiella pneumoniae* were resistant to Tetracycline (10 µg) [n = 3, 100.00%], Meropenem (10 µg) [n = 3, 100.00%], Cefuroxime (30 µg) [n = 3, 100.00%] and Vancomycin (30 µg) [n = 3, 100.00%] and Cotrimoxazole (25 µg) [n = 3, 100.00%]. More so, 66.67% [n = 2] of the *Klebsiella pneumoniae* isolates were resistant to Chloramphenicol (10 µg). Table 5 provides details on the resistance profile of *Escherichia coli* and *Klebsiella pneumoniae* against a panel of Gram-negative antibiotics.

**Table 5:** Resistance profile of Gram-negative isolates against Gram-negative antibiotic panel.

Antibiotics	*E.* *coli* [N = 1]	*K. pneumoniae* [N = 3]	*P.* *mirabilis* [N = 0]	*P.* *aeruginosa* [N = 0]
Tetracycline (10 µg)	1 [100.00]	3 [100.00]	─	─
Meropenem (10 µg)	1 [100.00]	3 [100.00]	─	─
Cefuroxime (30 µg)	1 [100.00]	3 [100.00]	─	─
Vancomycin (30 µg)	1 [100.00]	3 [100.00]	─	─
Amikacin (30 µg)	–	1 [33.33]	─	─
Cotrimoxazole (25 µg)	–	3 [100.00]	─	─
Gentamicin (10 µg)	–	1 [33.33]	─	─
Ampicillin (10 µg)	–	1 [33.33]	─	─
Ciprofloxacin (5 µg)	–	1 [33.33]	─	─
Ceftriaxone (30 µg)	–	1 [33.33]	─	─
Chloramphenicol (10 µg)	–	2 [66.67]	─	─

*Values reported are number of resistant isolates and proportion of the total number of isolates tested against the antibiotic [N]; “–” means isolate not tested against the antibiotic.*

#### Resistance profile of bacteria isolate against UTI antibiotic panel.

All seven bacterial pathogens reported in this study were isolated in urine samples as shown in Table 6. *Escherichia coli* was isolated in 41 urine samples, 92.68% [n = 38] were resistant to Norfloxacin and 80.49% [n = 33] were resistant to Ceftazidime. More so, 85.37% [n = 35], 87.80% [n = 36], 75.61% [n = 31], 60.98% [n = 25] and 60.98% [n = 25] of the *Escherichia coli* tested against Augmentin (30 µg), Nalidixic acid, Tetracycline, Piperacillin and Ceftriaxone respectively were resistant. *Klebsiella pneumoniae* was susceptible to the action of Amikacin. However, all the *Klebsiella pneumoniae* isolates tested against Augmentin (30 µg) [n = 16, 100.00%], Nalidixic acid [n = 16, 100.00%] were resistant as shown in Table 6. Furthermore, all the *Proteus mirabilis* isolates were resistant to Norfloxacin [n = 10, 100.00%] and Nalidixic acid [n = 10, 100.00%] but susceptible to Gentamicin [n = 10, 100.00%], Ceftriaxone [n = 0, 0.00%], Levofloxacin [n = 0, 0.00%] and Piperacillin [n = 0, 0.00%]. As depicted in Table 6, all isolated *Pseudomonas aeruginosa* were resistant [n = 2, 100.00%] to Norfloxacin, Ceftazidime, Nitrofurantoin, Augmentin (30 µg), Tetracycline, Piperacillin and Nalidixic acid. However, the isolates were also susceptible to Ciprofloxacin [n = 0, 0.00%] and Levofloxacin [n = 0, 0.00%]. Also, all the *Enterococcus faecalis* isolated from urine specimen were resistant to Norfloxacin [n = 2, 100.00%], Ceftazidime [n = 2, 100.00%], Nitrofurantoin [n = 2, 100.00%], Tetracycline [n = 2, 100.00%] and Nalidixic acid [n = 2, 100.00%]. Table 6 provides details on the resistance profile of the bacterial isolates in urine.

**Table 6:** Resistance profile of bacteria isolates against UTI antibiotic panel.

Antibiotics	Gram Negative				Gram Positive		
	*E.* *coli* [N = 41]	*K. pneumoniae* [N = 16]	*P. mirabilis* [N = 10]	*P. aeruginosa* [N = 2]	*E. faecalis* [N = 2]	*S. aureus* [N = 10]	*S. pneumoniae* [N = 1]
Ciprofloxacin	19 [46.34]	9 [56.25]	2 [20.00]	–	–	5 [50.00]	–
Norfloxacin	38 [92.68]	15 [93.75]	10 [100.00]	2 [100.00]	2 [100.00]	7 [70.00]	–
Ceftazidime	33 [80.49]	12 [75.00]	3 [30.00]	2 [100.00]	2 [100.00]	7 [70.00]	1 [100.00]
Amikacin	8 [19.51]	–	2 [20.00]	1 [50.00]	–	1 [10.00]	0 [0.00]
Nitrofurantoin	22 [53.66]	6 [37.50]	8 [80.00]	2 [100.00]	2 [100.00]	4 [40.00]	–
Gentamicin	14 [34.15]	5 [31.25]	–	1 [50.00]	–	1 [10.00]	–
Ceftriaxone	25 [60.98]	7 [43.75]	–	1 [50.00]	–	7 [70.00]	–
Levofloxacin	15 [36.59]	4 [25.00]	–	–	–	2 [20.00]	–
Augmentin	35 [85.37]	16 [100.00]	8 [80.00]	2 [100.00]	–	8 [80.00]	1 [100.00]
Tetracycline	31 [75.61]	13 [81.25]	6 [60.00]	2 [100.00]	2 [100.00]	8 [80.00]	–
Piperacillin	25 [60.98]	6 [37.50]	–	2 [100.00]	–	2 [20.00]	–
Nalidixic acid	36 [87.80]	16 [100.00]	10 [100.00]	2 [100.00]	2 [100.00]	9 [90.00]	1 [100.00]

*Values reported are number of resistant isolates and proportion of the total number of isolates tested against the antibiotic [N]; “–“: data value is zero.*

### Multidrug resistance profile of bacterial isolates

Out of 97 specimens with bacterial growth (individual isolates), 95 were multidrug resistant, thus representing a prevalence of 97.94% [CI_95_: 93.60 – 99.67] (Table 7). The prevalence of multidrug resistance among bacteria isolated from females was 98.72% [77/78, CI_95_: 93.15 – 99.69] and in males the prevalence of multidrug resistant bacteria was 94.74% [18/19, CI_95_: 75.13 – 98.77]. As shown in Table 7, gender was not significantly associated with multidrug resistance [p = 0.278]. All the bacteria pathogens isolated from specimen submitted by participants aged ≥ 60 years were multidrug resistant [100.00%, CI_95_: 71.69 – 100.00] (Table 7). More so, 98.00% [49/50, CI_95_: 89.55 – 99.52] and 97.44% [38/39, CI_95_:86.84 – 99.39] of the bacterial pathogens isolated in specimen submitted by participants aged ≤ 30 years and 31 – 59 years respectively were multidrug resistant as shown in Table 7. Statistically, age was not significantly associated with multidrug resistance [p = 0.883]. As shown in Table 7, type of bacteria isolate was significantly associated with multidrug resistance [p = 0.021]. Furthermore, all *Enterococcus faecalis* 100.00% [2/2, CI_95_: 36.84 – 100.00]*, Escherichia coli* 100.00% [42/42, CI_95_: 93.27 – 100.00]*, Klebsiella pneumoniae* 100.00% [19/19, CI_95_: 86.09 – 100.00]*, Proteus mirabilis* 100.00% [10/10, CI_95_: 76.16 – 100.00]*, Pseudomonas aeruginosa* 100.00% [2/2, CI_95_: 36.84 – 100.00] and *Streptococcus pneumoniae* 100.00% [1/1, CI_95_: 22.36 – 100.00] isolated from the specimens analysed were multidrug resistant (Table 7). However, 90.48% [19/21, CI_95_: 73.48 – 98.33] of the *Staphylococcus aureus* isolated were multidrug resistant.

**Table 7:** Distribution of multidrug resistance among participants and isolates.

	N	n	[%, CI_95_]	p-value
**Gender**				0.278
Male	19	18	94.74 [75.13 – 98.77]	
Female	78	77	98.72 [93.15 – 99.69]	
**Age group (years)**				0.883
≤ 30	50	49	98.00 [89.55 – 99.52]	
31 – 59	39	38	97.44 [86.84 – 99.39]	
≥ 60	8	8	100.00 [71.69 – 100.00]	
**Bacteria Isolate**				**0.021**
*Enterococcus faecalis*	2	2	100.00 [36.84 – 100.00]	
*Escherichia coli*	42	42	100.00 [93.27 – 100.00]	
*Klebsiella pneumoniae*	19	19	100.00 [86.09 – 100.00]	
*Proteus mirabilis*	10	10	100.00 [76.16 – 100.00]	
*Pseudomonas aeruginosa*	2	2	100.00 [36.84 – 100.00]	
*Staphylococcus aureus*	21	19	90.48 [73.48 – 98.33]	
*Streptococcus pneumoniae*	1	1	100.00 [22.36 – 100.00]	
**Total**	**97**	**95**	**97.94 [93.60 – 99.67]**	

*Values reported are the total number of participants in the subcategory [N], number positive for multidrug resistance or multidrug resistant isolate [n], prevalence of multidrug resistance [%] and its corresponding 95% confidence interval [CI*_*95*_*]*.

### Prevalence of extended-spectrum beta-lactamase producing enterobacteriaceae

The prevalence of ESBL producing isolates in this study was 76.06% [54/71, CI_95_: 64.91 – 84.46] (Table 8). Among the male participants, the prevalence of ESBL producing bacteria isolated from specimen submitted from this group was 84.61% [11/13, CI_95_: 57.19 – 95.34] and within the female cohort, the prevalence reported was 74.13% [43/58, CI_95_: 61.56 - 83.34] (Table 8). Statistically, gender was not significantly associated with the prevalence of ESBL producing bacteria [p = 0.431]. Furthermore, the prevalence of ESBL producing isolates was 85.71% [6/7, CI_95_: 47.35 – 96.81], 80.77% [21/26, CI_95_: 61.92 – 91.38] and 71.05% [27/38, CI_95_: 55.13 – 82.98] among participants aged ≥* *60 years, 31 – 59 years and ≤ 30 years respectively (Table 8). Also, age was not significantly associated with the prevalence of ESBL producing bacteria [p = 0.288]. The ESBL producing *Enterobacteriaceae* identified in this study were *Escherichia coli, Klebsiella pneumoniae* and *Proteus mirabilis* (Table 8). Of the 42 *Escherichia coli* isolated in this study, 38 were ESBL producing [90.47%, CI_95_: 77.86 – 96.11], while 13 out of the 19 isolated *Klebsiella pneumoniae* [68.42%, CI_95_: 45.72 – 84.61] also produced ESBL (Table 8). More so, 30.00% [3/10, CI_95_: 10.92 – 60.97] of the isolated *Proteus mirabilis* produced ESBL. As shown in Table 8, the type of bacteria pathogen isolated was significantly associated with the prevalence of ESBL production [p < 0.001].

**Table 8:** Distribution of ESBL producing Enterobacteriaceae.

	N	n	[%, CI_95_]	p-value
**Gender**				0.431
Male	13	11	84.61 [57.19 - 95.34]	
Female	58	43	74.13 [61.56 - 83.34]	
**Age group (years)**				0.288
≤ 30	38	27	71.05 [55.13 - 82.98]	
31 – 59	26	21	80.77 [61.92 - 91.38]	
* ≥ 60*	7	6	85.71 [47.35 - 96.81]	
**Bacteria Isolate**				**<0.001**
*Escherichia coli*	42	38	90.47 [77.86 - 96.11]	
*Klebsiella pneumoniae*	19	13	68.42 [45.72 - 84.61]	
*Proteus mirabilis*	10	3	30.00 [10.92 - 60.97]	
**Total**	**71**	**54**	**76.06 [64.91 - 84.46]**	

*Values reported are the total number of participants in the subcategory [N], number positive for ESBL production or producing isolates [n], prevalence of ESBL [%] and its corresponding 95% confidence interval [CI*_*95*_*]*.

## Discussion

This study examined the epidemiology and resistance profile of bacterial pathogens among patients receiving healthcare at the Shalina Health Centre in Accra, Ghana. It builds on previous research [19] by providing updated prevalence data and resistance patterns in a primary healthcare setting. Unlike earlier studies that focused on tertiary hospitals, our study presents AMR trends in a primary healthcare facility, a setting that is often underrepresented in AMR studies. The findings show a prevalence of bacterial infections [62.20%], consistent with previous reports from Ghana and other African settings [19–22]. The observed prevalence is also higher than previously reported figures, emphasizing the need for targeted infection control interventions in similar settings [23–26].

The predominance of Gram-negative bacteria [75.26%] over Gram-positive bacteria [24.74%] is consistent with global trends in healthcare-associated infections [27–29], where Gram-negative organisms are often more resistant due to their intrinsic mechanisms and adaptability [11,22,24,30,31]. These findings point to the importance of strengthening infection prevention and control (IPC) measures, including hand hygiene compliance, routine microbiological surveillance, and antimicrobial stewardship programs (ASP) to reduce the spread of resistant pathogens.

Most AMR studies in Ghana focus on large referral hospitals [23,24,32], leaving gaps in data from primary healthcare facilities, where many patients first seek treatment. Our findings confirm that resistance is widespread even at the primary care level, reinforcing concerns that resistant infections are not confined to major hospitals.

The predominance of *Escherichia coli*, *Staphylococcus aureus*, and *Klebsiella pneumoniae* aligns with global AMR trends, particularly in low-resource healthcare facilities [13,33]. The high resistance rates to fluoroquinolones, beta-lactams, and cephalosporins reflect similar patterns observed in West African hospitals [11,24,34–36]. As such, our results suggest there is an urgent need for revised empirical treatment guidelines that account for local resistance trends.

*Escherichia coli* was the most common pathogen, particularly in urinary tract infections (UTIs), followed by *Klebsiella pneumoniae* and *Proteus mirabilis*. This is consistent with its role as a major UTI pathogen, as reported in other studies [33,37,38]. The predominance of these pathogens suggests the need for enhanced hospital-acquired infection surveillance, particularly in outpatient and emergency settings where empirical antibiotic therapy is frequently used. Given that urine samples accounted for most positive bacterial cultures, targeted urinary catheter management protocols and education on proper hygiene should be emphasized to prevent recurrent infections. The high resistance rates to fluoroquinolones, beta-lactams, and cephalosporins reflect similar patterns observed in West African hospitals [11,24], further necessitating the urgent need for revised empirical treatment guidelines that account for local resistance trends.

The study found a significantly higher prevalence of bacterial infections in females (69.64%) compared to males (43.18%), which is consistent with findings from previous studies [25,39]. This difference may be linked to anatomical and physiological factors that make women more susceptible to UTIs, along with healthcare-seeking behaviours [40]. The absence of statistically significant age-related differences in infection rates suggests that infection control strategies should be broad-based, covering all demographic groups rather than being age specific. However, the finding that all isolates from older adults (≥ 60 years) were multidrug-resistant (MDR) highlights the need for targeted infection prevention interventions for high-risk populations, particularly elderly patients who may have prolonged antibiotic exposure due to chronic conditions [41,42].

The findings of this study are consistent with AMR trends observed in Ghana and across West Africa. The high resistance rates among *Escherichia coli* and *Klebsiella pneumoniae* reflect patterns seen in previous studies conducted in Ghanaian hospitals [22–24,34,35]. However, resistance levels in this primary healthcare setting are comparable to those reported in referral hospitals, suggesting that AMR is not restricted to facilities with frequent antibiotic exposure.

Ghana’s National Action Plan on AMR emphasizes the need for surveillance across different healthcare levels [43], but most surveillance efforts focus on tertiary institutions. This study contributes to a more comprehensive understanding of resistance trends by providing data from a setting that has been underrepresented in national reports. The high proportion of extended-spectrum beta-lactamase (ESBL)-producing Enterobacteriaceae indicates a growing challenge that may require adjustments to national treatment guidelines. Given the widespread resistance observed, empirical prescribing policies in Ghanaian healthcare settings may need revision to ensure first-line therapies remain effective.

This study found a high prevalence of MDR bacteria [97.94%], with all isolates of *Escherichia coli*, *Klebsiella pneumoniae*, and Proteus mirabilis showing resistance to multiple antibiotics. This raises serious concerns about antimicrobial resistance (AMR) and the need for local antimicrobial stewardship interventions [44,45]. Resistance to carbapenems such as meropenem, often considered last-resort antibiotics, is particularly concerning as it suggests the emergence of carbapenemase-producing bacteria, a growing global health threat [46,47]. The detection of extended-spectrum beta-lactamase (ESBL)-producing bacteria in 76.06% of Enterobacteriaceae further complicates treatment options, necessitating revised empirical antibiotic guidelines to curb inappropriate antibiotic use [21,32].

This study also demonstrates that multidrug-resistant bacteria are highly prevalent in primary care settings, an issue that has not received adequate attention. The fact that nearly all isolates exhibited resistance to multiple antibiotics raises concerns about treatment effectiveness in these facilities. Given that primary healthcare centres often prescribe empirical treatments due to limited diagnostic capabilities [48,49], understanding resistance patterns at this level is essential for improving antibiotic selection and patient outcomes.

Addressing AMR requires targeted interventions that go beyond routine surveillance. The results suggest an urgent need to strengthen antimicrobial stewardship programs (ASP) in primary healthcare facilities. This could be achieved through restricting the use of certain antibiotics in outpatient settings to slow the development of resistance. Given the high levels of resistance to commonly prescribed antibiotics, healthcare providers in these settings should be encouraged to use culture-based prescriptions whenever feasible, rather than relying solely on empirical treatment.

Improving diagnostic capacity is another critical step. Many primary healthcare centres lack the resources for routine microbiological testing, leading to overuse of broad-spectrum antibiotics. Establishing point-of-care diagnostic tools could allow clinicians to make more informed prescribing decisions. Additionally, patient education on antibiotic use should be integrated into routine consultations to reduce self-medication, which remains a major driver of resistance.

Healthcare facilities should also adopt infection prevention measures tailored to resource-limited settings. Strengthening hand hygiene protocols, ensuring proper sterilization of medical instruments, and improving sanitation within healthcare centres can help limit the spread of resistant bacteria. Given that urinary tract infections accounted for most resistant infections in this study, efforts should be made to improve catheter management and promote non-antibiotic preventive measures such as increased hydration and proper hygiene practices.

The findings of this study provide useful information for optimizing empirical treatment protocols in Ghanaian healthcare settings. Given the high resistance rates observed in *Escherichia coli* and *Klebsiella pneumoniae*, first-line treatments for UTIs and bloodstream infections should be reassessed to include alternative antibiotics with proven efficacy against MDR pathogens. The widespread resistance to fluoroquinolones and cephalosporins suggests that clinicians should consider carbapenem-sparing regimens where possible to maintain the effectiveness of last-resort antibiotics.

Although this study was conducted in a single healthcare facility, its findings are relevant to healthcare settings in Ghana and other low-resource environments, as similar AMR trends have been reported in other regional studies [34,50,51]. Expanding surveillance beyond tertiary hospitals and integrating antimicrobial stewardship at all levels of care will be necessary to slow the progression of drug-resistant infections in Ghana.

### Limitations of the study

This study relied solely on phenotypic techniques for bacterial species identification and extended-spectrum beta-lactamase (ESBL) confirmation, as molecular methods (e.g., PCR for *blaCTX*-M, *blaSHV*, *blaTEM* genes) were not employed due to resource constraints. Similarly, multidrug-resistant (MDR) isolates were not further characterized using molecular techniques such as PCR or whole-genome sequencing, which would have provided deeper insights into the specific genetic determinants of resistance.

While phenotypic testing remains a widely accepted approach in clinical microbiology and aligns with Clinical and Laboratory Standards Institute (CLSI) guidelines, molecular validation offers greater specificity and could further strengthen the findings. Future studies should incorporate genotypic resistance profiling to confirm MDR mechanisms, track resistance gene dissemination, and improve antimicrobial stewardship strategies.

## Conclusion

This study highlights the concerning epidemiology and resistance profiles of bacterial pathogens in a primary healthcare setting, revealing a high prevalence of infections, particularly among women, and an alarming rate of multidrug resistance (MDR), especially in older patients. The widespread resistance of *Staphylococcus aureus* to key antibiotics, alongside the predominance of extended-spectrum beta-lactamase (ESBL)-producing Enterobacteriaceae, underscores a growing threat to effective treatment and patient outcomes. These findings emphasize the urgent need for strengthened antimicrobial stewardship programs, improved infection prevention and control (IPC) strategies, and evidence-based antibiotic prescribing tailored to local resistance patterns. Given the high burden of MDR bacteria, targeted surveillance efforts and policy interventions are essential to mitigate the spread of resistant infections. Immediate action is required to preserve antibiotic efficacy, protect public health, and curb the rising burden of antimicrobial resistance (AMR) in primary healthcare settings.

## Supporting information

S1 Table Gram stain and biochemical test results. (XLSX)
